# Examining the Social and Mental Health Benefits of Virtual and In-Person Physical Activity Intervention Among Postsecondary Students: Quasi-Experimental Study

**DOI:** 10.2196/92076

**Published:** 2026-06-11

**Authors:** Melissa L deJonge, Amy E Nesbitt, Simon C Darnell, Chloe A Hamza, Catherine M Sabiston

**Affiliations:** 1 Faculty of Kinesiology and Physical Education, University of Toronto, 55 Harbord Street, Toronto, Ontario, M5S 2W6, Canada, 1 (416) 978-3436; 2 Rehabilitation Sciences Institute, University of Toronto, Toronto, Ontario, Canada; 3 Department of Applied Psychology and Human Development, University of Toronto, Toronto, Ontario, Canada

**Keywords:** social inclusion, mental health, exercise, lifestyle, young adult, social determinants of health

## Abstract

**Background:**

Physical activity (PA) is a promising prevention approach for supporting mental health and enhancing social inclusion among postsecondary students. However, it is unclear whether similar outcomes are realized when PA programming is delivered in-person versus virtually.

**Objective:**

Using data from a multiphase research project, the purpose of the study was to examine the influence of on-campus PA programming (virtual and in-person delivery) on mental ill health symptoms (ie, anxiety and depression), social inclusion indices (ie, social connectedness, emotional ties, and social relationship quality), and well-being. Three objectives were addressed: (1) to assess pre-post change in symptoms, social inclusion indices, and well-being for virtual and in-person delivery; (2) to evaluate whether outcome change over time differed by delivery mode; and (3) to examine whether change in symptoms and social inclusion indices predicted change in well-being for both delivery modes.

**Methods:**

Physically inactive postsecondary students experiencing mental ill health participated in a 6-week structured and supervised PA program. Pre-post intervention data were collected across 3 phases, and the analytical samples included: 1. In-person delivery (n=87; 82%, 69/84 young adults; 86%, 74/86 women; 38%, 33/86 White; 20%, 17/86 Chinese; 86%, 75/87 with mental illness; 2. Virtual delivery (n=62; 69%, 42/61 young adults; 95%, 59/62 women; 34%, 21/62 White; 21%, 13/62 South Asian; 55%, 34/62 with mental illness), and 3. Data from students who received in-person or virtual delivery: (n=92; 67%, 61/91 young adults; 90%, 83/92 women; 32%, 29/92 White; 20%, 18/92 South Asian; 59%, 54/92 with mental illness). Data were analyzed using 2-tailed paired samples *t* tests to address objective 1, a 2 (delivery mode) × 2 (time: pre-post) repeated-measures ANOVA to address objective 2, and hierarchical regression analyses to address objective 3.

**Results:**

Both virtual and in-person PA delivery were effective for symptom reduction and social inclusion improvements across all outcomes (*P*<.001), with moderate-to-large effects. There was no significant time × delivery mode (*F*_5,84_=0.72, *ηp*²=0.04, *P*=.60) interaction effect. Change in social inclusion indices explained unique variance in well-being, beyond covariates (gender, mental illness, and ethno-racial identity), and symptom reduction for virtual (*R*^2^*_adj_* = 0.75, *ΔR*^2^*=*0*.*08*, P<.*001) and in-person (*R*^2^*_adj_* = 0.72, *ΔR*^2^=0.16, *P*<.001) PA delivery.

**Conclusions:**

Online distance learning is increasing across postsecondary settings worldwide, underscoring the need for accessible, technology-enabled mental health prevention interventions. The results provide support for the effectiveness of virtual and in-person PA programming for reducing symptoms of anxiety and depression, while also enhancing social inclusion indices and overall well-being. Social inclusion indices were also a key contributor to improved well-being, emphasizing the relevance of social factors in both virtual and in-person PA-based mental health prevention strategies for postsecondary students.

## Introduction

### Background

Young adulthood is a distinct developmental period in the life course, typically defined as ages 18‐29 years, and characterized by identity exploration, instability, and self-focus [1]. Young adulthood represents the highest-risk period for the onset of mental illness and symptoms of mental ill health [2]. Postsecondary students are an important subgroup of this population, as they predominantly fall within the young adult age range and experience transition-related stressors (eg, academic pressure, adjustment to new social environments, and disruptions to health behaviors) that increase the risk of mental ill health [3-7]. Large-scale international survey data [8-10] indicate that a substantial proportion of students report symptoms of depression and anxiety, as well as challenges related to social inclusion (eg, loneliness) and engagement in health-risk behaviors (eg, low PA [physical activity] and poor sleep). In response, policy initiatives position postsecondary settings as critical contexts for supporting young adult mental health, given their potential to address behavioral and social determinants of mental health (eg, health behaviors and social connectedness) across campus life, institutional culture, and academic systems [11,12].

Postsecondary settings include universities, colleges, and other educational institutions offering formal learning opportunities beyond secondary education (eg, vocational schools) [13]. To address postsecondary student mental health, there is an international priority to integrate whole-campus approaches [11,12,14]. Whole-campus approaches extend beyond the provision of primary clinical care to include community-based prevention and early intervention services to target social, cultural, and structural influences on student mental health [11,12]. Evidence-based prevention and early intervention initiatives can be conceptually grounded within clinical staging models, which are increasingly implemented across global mental health contexts [15]. Clinical staging models represent mental health along a multidimensional continuum from health to illness, distinguishing early (subclinical) stages from later stages characterized by severe and persistent mental illness [15]. Mental health reflects the capacity to realize one’s potential, cope with stressors, and participate in society, whereas mental ill health refers to subclinical experiences of distress and impaired functioning, and mental illness captures more severe, persistent, and clinically diagnosable conditions [15,16]. Prevention and early intervention initiatives are often offered in the community during early clinical stages, where the purpose is to target social determinants of and risk factors for mental ill health (eg, social disconnection, health-risk behaviors, and distress) associated with the onset and progression of mental illness [15,17]. Yet, gaps in the implementation and scale-up of prevention strategies across postsecondary campuses are evident and include evidence-based programming approaches that are sustainable and accessible to students [18].

A promising, sustainable, and accessible community-based prevention approach is on-campus PA programming tailored toward student mental health [19,20]. On-campus PA programming has the potential to support whole-campus priorities, including social (eg, community engagement and social connection), structural (eg, accessible, inclusive PA spaces and referral pathways), and cultural (eg, reducing barriers to help-seeking) influences on student mental health [21,22]. Evidence for the effectiveness and feasibility of PA programs for student mental health and for complementing primary treatment options is growing [20,23]. This is supported by practice guidelines recommending PA as an alternative and complementary therapeutic approach to support physical health, reduce mental ill health symptoms, and promote mental health across clinical and nonclinical community-based settings [24,25]. Importantly, findings from a systematic review on PA interventions for postsecondary student mental health offer evidence of effectiveness on outcomes including depression, anxiety, and stress [23]. This body of literature highlights the importance of improving student access to support structures (eg, structured and tailored mental health PA programs) to encourage participation in PA as an evidence-based, therapeutic intervention for both clinical and nonclinical populations [23,26]. Improving PA support structures reflects a broader movement in mental health care, aimed at enhancing access to lifestyle-based therapeutic interventions [24]. Indeed, to better address postsecondary student mental health, researchers support a need for expanded treatment options, beyond traditional modalities (eg, therapy and medication), with a focus on evidence-informed prevention interventions offered in the community, such as PA programming [27,28]. Aligned with clinical staging models, PA interventions can be offered to promote well-being within the campus community, as well as integrated within the range of treatment approaches in campus mental health care, where care offered is proportionate to the stage of illness and guided by shared decision-making [15,24].

While research demonstrating the effectiveness of tailored PA programs for student mental health is growing, a limitation in the literature is the predominant focus on reducing mental ill health symptoms, with limited exploration of how such programs impact broader mental health outcomes, including social inclusion indices [23,26]. Social inclusion is defined to encompass a sense of belonging, connection, and possessing the means and opportunity to participate in valued social and community roles and activities [29]. Leisure activities, including community-based PA engagement, are supported as important for promoting postsecondary students’ mental health, community engagement, social support, and feelings of social connectedness and belonging [30-32]. In addition, extracurricular activities that involve PA have been identified by students as important interventions for supporting social interaction and belonging [28,33]. Intervention research, however, has yet to comprehensively examine the influence of structured PA programming on social inclusion indices (eg, social connectedness, social support, and belonging) among students, with current research predominantly being qualitative or cross-sectional study designs [23,26,34]. Assessing the effects of structured PA interventions on social inclusion indices is important for shifting PA research beyond symptom reduction toward a more holistic understanding of the mental health benefits of PA among postsecondary students.

Finally, the growth of online distance learning and hybrid learning formats across international settings can create accessibility barriers, limiting students’ ability to engage with on-campus mental health programs and community activities [35-37]. Researchers examining virtually delivered lifestyle interventions among individuals living with mental illness support their utility for facilitating engagement in health behavior change [38,39]. However, it remains unclear whether virtual and in-person PA delivery yield similar mental health prevention benefits, as systematic review findings support limited research on the effectiveness of virtual PA delivery for mental health and social outcomes [40,41]. Further understanding the effectiveness of both in-person and virtual PA delivery has important implications for expanding PA programs on postsecondary campuses, as digital technologies have been identified as critical for supporting student mental health [42]. Indeed, virtual PA programming has been supported as a postpandemic priority and may offer greater flexibility to students, reduce delivery costs, and expand reach to distance learners, a student population group at increased risk of social isolation and mental ill health [43].

### The Present Investigation

To address the gaps in the literature, data from a multiphase research project aimed at evaluating the effectiveness of a PA program (MoveU.HappyU) for postsecondary student mental health at the University of Toronto were analyzed in this study. By using a multiphase approach, a more comprehensive understanding can be gained of how PA programs impact mental ill health symptoms and social inclusion indices, and whether in-person and virtual delivery modes yield comparable outcomes. The overarching purpose of this study was to explore the influence of on-campus PA programming (virtual and in-person delivery) on mental ill health symptoms (ie, anxiety and depression), social inclusion indices (ie, social connectedness, emotional ties, and social relationship quality), and well-being. Three specific objectives were addressed: (1) to examine the direction and magnitude of pre-post change in mental ill health symptoms, social inclusion indices, and well-being; (2) to examine whether pre-post change in mental ill health symptoms, social inclusion indices, and well-being differ between virtual and in-person delivery; and (3) to examine the degree to which change in mental ill health symptoms and social inclusion indices predict change in well-being. Guided by social inclusion research [29], social well-being models [44], and previous research [45,46], it was hypothesized that (1) there would be significant mental ill health symptom reduction and improvements in social inclusion indices and well-being pre-post intervention; (2) there would be no difference in pre-post intervention change in mental ill health symptoms, social inclusion indices, and well-being between virtual and in-person program delivery; and (3) positive change in social inclusion indices would contribute unique variance to positive change in well-being compared to the contribution of mental ill health symptom reduction alone.

## Methods

### Study Design

MoveU.HappyU has been implemented at the University of Toronto since May 2015 and is an ongoing research initiative. Guided by the Obesity-Related Behavioral Intervention Trials model [47], adaptations have been made to support scale-up and broader implementation of the program across 3 data collection phases (Table 1). Phase 1 involved in-person delivery to students, while Phase 2 included virtual delivery during COVID-19 campus closures and in-person delivery after campuses reopened. Phase 3 returned to fully in-person delivery. Phases 1 and 2 were implemented using quasi-experimental designs, while Phase 3 was conducted as a randomized controlled trial (RCT), examining the effectiveness of one-on-one in-person PA and group-based PA on mental health and social outcomes compared to a waitlist control. For this study, analyses were restricted to the one-on-one in-person PA intervention arm. Full details of the RCT design are provided in the published protocol [48], including PA intervention delivery procedures and materials, which were consistent across the phases.

**Table 1.** MoveU.HappyU data collection phases and alignment of the phases with the research objectives and analytical samples.

Study characteristics	Phase 1 (in-person delivery)	Phase 2 (virtual delivery)	Phase 3 (in-person delivery)
Study design	Proof-of-concept pretest-posttest study design	Proof-of-concept pretest-posttest study design	A 3-arm parallel RCT^a^ assessing immediate and 1-month effects of in-person one-on-one and group physical activity vs a 10-week waitlist control
Years of implementation	March 2015-February 2020	November 2020-June 2022	October 2023-2025
Key outcomes	Well-being^b^ Depression^b^ Anxiety^b^ Emotional ties^b^ Social relationship quality^c^	Well-being^b^ Depression^d^ Anxiety^e^ Emotional ties^b^ Social connectedness^f^	Key measures are as outlined in Phase 2
Sample size	N_in-person_=87	N_virtual_=62 N_in-person_=5	Data from the one-on-one in-person delivery arm was used (N_in-person_=25)
Analytical samples and alignment with research objectives	Sample 1 (N_in-person_=87)*.* Used to address objectives 1 and 3	Sample 2 (N_virtual_=62). Used to address objectives 1 and 3	Sample 3 (N_in-person and virtual_=92) combined in-person delivery from Phase 2 and 3 (n=30) with virtual delivery from Phase 2 (n=62). Used to address objective 2

a RCT: randomized controlled trial.

b Mental Health Index-38 [49].

c Quality of Life Enjoyment and Satisfaction Questionnaire - Short Form [50].

d Patient Health Questionnaire-9 [51].

e Generalized Anxiety Disorder Questionnaire-7 [52].

f Social Connectedness Scale [53].

Specifically, all students received a 6-week, individually tailored, supervised PA program consisting of weekly 1-hour sessions. The 6-week duration is supported by evidence indicating that PA interventions of at least 4 weeks are effective for improving postsecondary student mental health, with moderate effects [26]. From a pragmatic perspective, the timeframe also allows for sufficient recruitment at the start of the semester to support program completion prior to final semester exams and academic breaks (eg, winter and summer holidays). Grounded in behavior change theory and empirical evidence on PA for mental health [46,54-56], each 1-hour session included 30 minutes of PA behavior change coaching and 30 minutes of PA training.

The analytical samples included 87 students for sample 1 (Phase 1 in-person delivery), 62 students for sample 2 (Phase 2 virtual delivery), and 92 students for sample 3, which included Phase 2 virtual delivery (n=62), Phase 2 in-person delivery (n=5), and Phase 3 in-person delivery (n=25). Students were unique to each phase, with no participant overlap across phases. To address objective 1, sample 1 and sample 2 were examined separately to understand program effectiveness for in-person and virtual delivery. To address objective 2, sample 3 was used to explore whether the mode of delivery influenced program effectiveness. To address objective 3, sample 1 and sample 2 were examined in separate models again to understand the unique influence of social inclusion indices on well-being, within in-person and virtual delivery.

### Study Setting and Delivery Context

Across the intervention phases, infrastructure provided by the Mental Health and Physical Activity Research Centre (MPARC) at the University of Toronto was used. In Phase 1, PA sessions were delivered by graduate students affiliated with MPARC. Program trainers were certified PA professionals with academic backgrounds in PA and health psychology. Training included a suicide prevention workshop, as well as graduate-level coursework in exercise psychology and behavior change coaching principles. In Phases 2 and 3, as the program was scaled, graduate students from MPARC and certified PA trainers from Sport and Recreation Services were engaged using a train-the-trainer model [57]. The research team collaborated with on-campus Sport and Recreation Services to develop and deliver standardized training focused on behavior change coaching and PA program delivery for mental health support.

For in-person delivery, sessions were delivered in private PA spaces in MPARC, conveniently located in the campus athletics and recreation center. Virtual delivery was implemented during campus closures related to the COVID-19 pandemic. Weekly sessions were delivered over secure, university-hosted Zoom (Zoom Video Communications) calls, with sessions tailored to students’ available space and equipment. Aside from delivery mode and setting, the intervention protocol (refer to the “Study Design” section) remained consistent across phases, with all sessions delivered synchronously.

### Participants and Procedures

Recruitment targeted undergraduate and graduate postsecondary students who were physically inactive and experiencing mental ill health. As the program evolved to support broader scalability, the recruitment and screening processes used to determine eligibility were refined accordingly.

In Phase 1, students were recruited through referrals from campus Health and Wellness Services, as well as through posters displayed in waiting areas at the campus mental health center. In alignment with campus mental health strategies promoting early access, self-referral, and the integration of nonclinical supports, broader recruitment strategies were implemented in Phases 2 and 3 [11,12,27]. Recruitment in Phases 2 and 3 occurred via outreach through Accessibility Services, Health and Wellness Services, and student life listservs. Digital recruitment materials (eg, email scripts and poster advertisements) provided information about the program and directed students to an online intake questionnaire. Interested participants completed an online intake questionnaire via a secure platform (REDCap [Research Electronic Data Capture]) [58] to initiate the intake process. In Phase 1, this questionnaire was used for scheduling purposes only, and eligibility was confirmed during an intake session, whereas in Phases 2 and 3, eligibility screening was conducted based on intake questionnaire responses, and eligible participants were invited to complete an intake session. During intake sessions, written informed consent was obtained, PA readiness was assessed, and baseline self-report measures were completed to assess demographic characteristics, PA, mental ill health symptoms, and social inclusion indices. Across intervention phases, recruitment occurred at the beginning of the winter, spring, and summer semesters, with participants starting the intervention in the early to midsemester period and finishing prior to the end of the semester.

In Phase 1, eligibility was determined based on clinical judgment by campus mental health care providers and established program criteria. Students were required to be physically inactive, defined as engaging in <150 minutes of moderate-to-vigorous physical activity (MVPA) per week in accordance with global PA guidelines [59], and to be seeking mental health support. In Phases 2 and 3, participants were screened as insufficiently active based on a score of ≤23 metabolic equivalents of MVPA per week on the Godin Leisure-Time Exercise Questionnaire [60], consistent with established classifications of insufficient PA and indicative of not meeting global recommended PA guidelines [61]. Mental ill health eligibility in Phase 2 was determined by the following criteria: (1) scoring at or above established clinical cutoffs for mild depression (Patient Health Questionnaire-9 [PHQ-9] score >10 [51]) or anxiety (Generalized Anxiety Disorder-7 [GAD-7] score >5 [52]); (2) screening positive for eating pathology (SCOFF Questionnaire score ≥2 [62]); (3) reporting a diagnosed mental illness; or (4) rating mental health as “poor” on a single-item 5-point scale (1=poor to 5=excellent). In Phase 3, rather than emphasizing symptom thresholds, inclusion criteria included rating mental health as “poor,” “fair,” or “good” on a single-item 5-point scale (1=poor to 5=excellent) and seeking PA for mental health promotion. This approach was adopted to align with the importance of intervening early, before symptoms escalate, to promote mental health and prevent mental ill health [15].

Exclusion criteria across all phases included: (1) sufficiently active, (2) unsuccessful PA clearance (Physical Activity Readiness Questionnaire for Everyone+ [63]), and (3) not being a postsecondary student seeking mental health support. Students were eligible to participate in the study regardless of whether they were using additional forms of mental health treatment or support (eg, therapy and medication).

Following the intake session, eligible participants were matched with a program trainer and worked with the same trainer for the 6-week duration for both virtual and in-person PA delivery. Participants completed an online self-report questionnaire at 2 time points (baseline: T1 and postintervention: T2). For purposes of this study, participants were included if they had complete data at 2 time points.

### Measures

Internal consistency of the measures was assessed and supported using an omega coefficient (*ω*) or a Spearman-Brown coefficient (*ρ*) for 2-item scales, with values greater than 0.70 [64].

#### Demographic Characteristics

Participants self-reported their age, PA engagement, gender, ethno-racial identity, past-year mental illness diagnosis, and past-year treatment among participants reporting a mental illness (refer to Table 2 for response options).

**Table 2.** Student demographic characteristics across analytical sample 1 (n=87), sample 2 (n=62), and sample 3 (n=92).

Demographic characteristics	Sample 1 (in-person)	Sample 2 (virtual)	Sample 3 (in-person and virtual)
Age (years), mean (SD)	22.96 (3.90)	24.57 (4.86)	24.81 (5.01)
Young adult, n/N (%)	69/84 (82)	42/61 (69)	61/91 (67)
Adult, n/N (%)	15/84 (18)	19/61 (31)	30/91 (33)
Participants, n	87	62	92
Baseline MVPA^a^, mean (SD)	28.25 (39.57)	45.90 (48.48)	51.52 (50.20)
Postintervention MVPA, mean (SD)	128.36 (88.79)	126.21 (105.04)	140.05 (100.31)
Gender, n/N (%)			
Women	74/86 (86)	59/62 (95)	83/92 (90)
Nonbinary	0/86 (0)	3/62 (5)	5/92 (5)
Men	12/86 (14)	0/62 (0)	4/92 (4)
Ethno-racial identity, n/N (%)			
White	33/86 (38)	21/62 (34)	29/92 (32)
Chinese	17/86 (20)	10/62 (16)	17/92 (19)
South Asian (eg, East Indian, Pakistani, and Sri Lankan)	9/86 (11)	13/62 (21)	18/92 (20)
Japanese	0/86 (0)	0/62 (0)	1/92 (1)
Arab	2/86 (2)	0/62 (0)	0/92 (0)
Black	2/86 (2)	2/62 (3)	4/92 (4)
Filipino	1/86 (1)	0/62 (0)	0/92 (0)
Latin American	4/86 (5)	1/62 (2)	2/92 (2)
Southeast Asian (eg, Cambodian, Indonesian, Laotian, and Vietnamese)	1/86 (1)	2/62 (3)	2/92 (2)
West Asian (eg, Afghan, Iranian, and Syrian)	3/86 (4)	3/62 (5)	4/92 (4)
Korean	1/86 (1)	3/62 (5)	4/92 (4)
Not listed	4/86 (5)	1/62 (2)	2/92 (2)
Multiple selected groups	9/86 (11)	6/62 (10)	9/92 (10)
Mental illness^b^, n/N (%)			
Diagnosis	75/87 (86)	34/62 (55)	54/92 (59)
No diagnosis	12/87 (14)	28/62 (45)	38/92 (41)
Anorexia	0/87 (0)	1/62 (2)	2/92 (2)
Anxiety	58/87 (67)	29/62 (47)	49/92 (53)
Attention deficit hyperactivity disorder	7/87 (8)	0/62 (0)	9/92 (10)
Bipolar disorder	2/87 (2)	1/62 (2)	1/92 (1)
Bulimia	2/87 (2)	2/62 (3)	2/92 (2)
Depression	63/87 (72)	20/62 (32)	34/92 (37)
Insomnia	10/87 (12)	7/62 (11)	12/92 (13)
Other sleep disorders	5/87 (6)	2/62 (3)	3/92 (3)
Obsessive-compulsive disorder	8/87 (9)	4/62 (7)	5/92 (5)
Panic disorder	23/87 (26)	11/62 (18)	16/92 (17)
Phobia	4/87 (5)	2/62 (3)	4/92 (4)
Substance abuse or addiction (eg, alcohol or other drugs)	3/87 (3)	0/62 (0)	0/92 (0)
Other addictions (eg, gambling, internet, and sexual)	1/87 (1)	1/62 (2)	1/92 (1)
Other mental illness	12/87 (14)	4/62 (7)	7/92 (8)
Mental illness treatment^c^, n/N (%)			
No treatment	13/75 (17)	6/34 (18)	13/54 (24)
Treatment	62/75 (83)	28/34 (82)	41/54 (76)
Treatment type, n/N (%)			
Medication	12/62 (19)	10/28 (36)	13/41 (32)
Therapy	17/62 (27)	6/28 (21)	10/41 (24)
Medication and therapy	31/62 (50)	11/28 (39)	16/41 (39)
Other	2/62 (3)	1/28 (4)	2/41 (5)

a MVPA: moderate-to-vigorous physical activity.

b Percentages do not add up to 100% because students could select more than one response option.

c Reported among students experiencing a diagnosed mental illness.

#### Physical Activity

Weekly MVPA was reported at baseline and postintervention to assess whether students were meeting global PA guidelines linked to health and well-being [59,61]. For descriptive purposes, the average weekly minutes of MVPA are reported at T1 and T2. In Phase 1 and Phase 2, the International Physical Activity Questionnaire - Short Form was used [65]. For reasons related to ease of administration and scoring, the Godin Leisure-Time Exercise Questionnaire was used in Phase 3 [60]. A common modification of assessing the average duration (in minutes) per session of each intensity category of PA was included to assess average weekly minutes of MVPA [61].

#### Depression Symptoms

For Phase 1 data collection, the 4-item subscale from the Mental Health Index-38 (MHI-38 [49]) was used. Students were asked to report past-month depression symptoms on a 6-point Likert scale for 3 items ranging from 1 (all of the time) to 6 (none of the time) or a 5-point Likert scale for one item ranging from 1 (yes, to the point that I did not care about anything for days at a time) to 5 (no, never felt depressed at all). Items were summed and reverse-scored, where higher scores reflect greater depression symptoms. For Phases 2 and 3, the PHQ-9 [51] was used. This change in the self-report survey reflected a shift in measurement priorities to align with survey methodologies used by Health and Wellness Services at the University of Toronto. The PHQ-9 measures the presence and severity of depression symptoms over the past 2 weeks on a 4-point Likert scale ranging from 0 (not at all) to 3 (nearly every day), where higher scores indicate greater depression symptoms. Estimates of internal consistency for the analytical samples were: sample 1 (*ω*_pre_=0.87 and *ω*_post_=0.90), sample 2 (*ω*_pre_=0.83 and *ω*_post_=0.90), and sample 3 (*ω*_pre_=0.80 and *ω*_post_= 0.88).

#### Anxiety Symptoms

For Phase 1 data collection, the 9-item subscale from the MHI-38 [49] was used. Students were asked to report past-month anxiety symptoms on a 6-point Likert scale ranging from 1 (all of the time) to 6 (none of the time). Items were summed and reverse-scored, where higher scores reflect greater anxiety symptoms. For Phases 2 and 3, the GAD-7 [52] was used. Similar to the shift in measurement for depression symptoms, the use of the GAD-7 was adopted to align with the survey methodologies used by Health and Wellness Services across the University of Toronto. The GAD-7 measures the frequency and severity of symptoms associated with anxiety during the past 2 weeks, ranging from 0 (not at all) to 3 (nearly every day), where higher scores indicate greater anxiety symptoms. Estimates of internal consistency for the analytical samples included: sample 1 (*ω*_pre_=0.89 and *ω*_post_=0.93), sample 2 (*ω*_pre_=0.87 and *ω*_post_=0.91), and sample 3 (*ω*_pre_=0.85 and *ω*_post_=0.90).

#### Social Relationship Quality

For Phase 1, a single item (ie, taking everything into consideration, during the past week how satisfied have you been with your social relationships) from the Quality of Life Enjoyment and Satisfaction Questionnaire - Short Form [50] was used to assess past-week social relationship quality on a 5-point Likert scale ranging from 1 (very poor) to 5 (very good), with higher scores indicating better social enjoyment and satisfaction. In alignment with the growing priority on postsecondary campuses to address student belonging and connectedness through mental health research [28], Phases 2 and 3 assessed social connectedness instead of social relationship quality.

#### Social Connectedness

For Phases 2 and 3, the 8-item Social Connectedness Scale was used [53]. Items reflect emotional distance and lack of connection with others and are rated on a 6-point Likert scale ranging from 1 (strongly agree) to 6 (strongly disagree). Higher scores indicate greater perceived social connectedness and belonging, with total scores ranging from 8 to 48. Estimates of internal consistency for the analytical samples were: sample 2 (*ω*_pre_=0.94 and *ω*_post_=0.96) and sample 3 (*ω*_pre_=0.93 and *ω*_post_=0.95).

#### Emotional Ties

In all intervention phases, the 2-item subscale from the MHI-38 [49] was used to measure the strength and quality of an individual’s relationship with others on a 6-point Likert scale ranging from 1 (all of the time) to 6 (none of the time). Items were summed and reverse-scored, where higher scores reflect greater emotional ties. Estimates of internal consistency for the analytical samples were sample 1 (*ρ*_pre_=0.83 and *ρ*_post_=0.86), sample 2 (*ρ*_pre_=0.84 and *ρ*_post_=0.74), and sample 3 (*ρ*_pre_=0.85 and *ρ*_post_=0.78).

#### Well-Being

In all intervention phases, the 14-item well-being subscale from the MHI-38 [49] was used. Students were asked to report how often during the past month they experienced well-being symptoms on a 6-point Likert scale ranging from 1 (all of the time) to 6 (none of the time). Items were reverse-scored, and a total summed score was used, where higher scores represent more positive experiences of well-being. Estimates of internal consistency for the analytical samples were sample 1 (*ω*_pre_=0.89 and *ω*_post_=0.93), sample 2 (*ω*_pre_=0.91 and *ω*_post_=0.92), and sample 3 (*ω*_pre_=0.89 and *ω*_post_=0.91).

### Data Analysis

The data were screened for outliers using z-scores (|z|> 3.00 SD), missing data, and assumptions of regression analyses [66] and ANOVAs [67]. Descriptive statistics, including means, SDs, and frequency counts, were conducted to describe the samples. For primary analyses, demographic variables were dichotomized to simplify interpretation and based on data distributions. Gender was dichotomized as women (1) and nonwomen (0), age as young adults (18‐25 y; 1) and adults (26‐50 y; 0), ethno-racial identity as White (1) and less represented ethno-racial groups (0), and mental illness as reported past-year diagnosis (1) and no reported past-year diagnosis (0).

To address objective 1, 2-tailed paired samples *t* tests were conducted to examine pre-post change in mental ill health symptoms, social inclusion indices, and well-being. Bonferroni correction was applied to control for multiple comparisons across a family of 5 tests (Bonferroni-adjusted significance level, *α*=.01). To address objective 2, and test for equivalency, between-group differences (delivery mode: virtual and in-person delivery) in baseline main study outcomes and dichotomized demographic characteristics were examined using chi-square tests and independent samples *t* tests. For the main analysis, a 2 (delivery mode: virtual and in-person) × 2 (time: T1 and T2) repeated-measures ANOVA was conducted to examine pre-post change in the intervention outcomes and to examine differences in change between virtual and in-person program delivery. To address objective 3, bivariate correlations were conducted to examine associations between change in primary outcomes using standardized residual change scores and dichotomized demographic variables, with Spearman correlations used for analyses involving dichotomous variables and Pearson correlations for continuous variables. Standardized residual change scores were calculated for social inclusion indices, mental ill health symptoms, and well-being by regressing postintervention (T2) scores on the baseline (T1) counterparts and saving the residuals. Hierarchical regression analyses were then conducted using standardized residual change scores. Given that the purpose of this research was to explore whether change in social inclusion indices explained additional unique variance in well-being than change in mental ill health symptoms, covariates were included in Step 1 of the hierarchical model, mental ill health symptoms were included in Step 2, and social inclusion indices were entered in Step 3.

Effect sizes were calculated to reflect the magnitude of the associations in the hierarchical linear regression model using standardized beta coefficients (*β*), to capture the magnitude of pre-post change using Cohen *d*, reported as Cohen *d_z_* to reflect within-subject effects for the paired samples *t* tests, and partial eta squared (*ηp*²) for the ANOVA. Based on empirically derived guidelines in social psychology, Cohen *d* and *β* values of 0.15, 0.36, and 0.65 were interpreted as small, moderate, and large effects [68,69]. *ηp²* values of 0.01, 0.06, and 0.14 were indicative of small, moderate, and large effects, respectively [70]. All analyses were conducted using IBM SPSS version 28.0.1.0 (142).

### Ethical Considerations

Study design and procedures were approved by the University of Toronto Health Sciences Research Ethics Board (Phase 1 and Phase 2 protocol number: 31097; Phase 3 protocol number: 45228). Written informed consent was obtained from all participants prior to study enrollment. As part of the consent process, participants were provided with a detailed explanation of the study objectives, the voluntary nature of participation, their right to withdraw, and the potential risks and benefits of participation. Participant confidentiality was protected throughout the study. Data were deidentified and replaced with participant identification codes, and all data were reported in aggregate form. Any information linking identifying information to study identification codes was stored separately from research data and accessible only to authorized members of the research team. Across intervention phases, all students who expressed interest in the program, regardless of eligibility, were provided with information about available campus and community mental health and support services. Program PA trainers and staff were also trained to identify and respond to participants experiencing distress and to facilitate access to appropriate supports where needed.

## Results

### Participants

Participant flow for the data collection phases (Phase 1, Phase 2, and Phase 3), including adherence and reasons for dropout can be found in Figures S1-S3 in Multimedia Appendix 1. Participant demographic characteristics for the analytical samples are presented in Table 2.

### Preliminary Findings

Missing value analyses at the case-level and item level were conducted separately across all 3 analytical samples. Data were missing at random as suggested by Little’s Missing Completely at Random Test for sample 1 (*χ*^2^_895_=810.00, *P*=.98), sample 2 (*χ*^2^_574_=1.26, *P*≥.99), and sample 3 (*χ*^2^_361_=359.83, *P*=.51). Missing data at the item-level were low (<4%) and main study variables were handled using item-level median imputation and demographic variables were handled using list-wise deletion. Skewness (±2.00) and kurtosis (±7.00) values indicated a normal distribution of the data across the main study variables.

### Main Findings

#### Objective 1: Pre-Post Intervention Changes in Mental Ill Health Symptoms and Social Inclusion Indices

##### In-Person Delivery

There was a significant increase in well-being postintervention (mean 47.38, SD 13.19) compared to preintervention (mean 37.71, SD 10.34; *t*_86_=7.35; Cohen *d*_z_=0.78, 95% CI 0.55-1.03; *P*<.001), emotional ties postintervention (mean 7.87, SD 2.72) compared to preintervention (mean 6.70, SD 2.67; *t*_86_=4.95; Cohen *d_z_*=0.53, 95% CI 0.30-0.75; *P*<.001), and social relationship quality postintervention (mean 3.48, SD 1.03) compared to preintervention (mean 2.95, SD 1.12; *t*_86_=3.87; Cohen *d_z_*=0.42, 95% CI 0.20-0.63; *P*<.001). There was also a significant reduction in anxiety symptoms postintervention (mean 29.10, SD 9.66) compared to preintervention (mean 34.43, SD 8.66; *t*_86_=−6.68; Cohen *d_z_*=−0.72, 95% CI −0.95 to −0.48; *P*<.001) and depression symptoms postintervention (mean 11.89, SD 4.18) compared to preintervention (mean 14.96, SD 4.04; *t*_86_=−6.33; Cohen *d_z_=*−0.68*,* 95% CI −0.91 to −0.44*; P*<.001). A medium within-subjects effect size was demonstrated for pre-post change in social relationship quality and emotional ties, and a large effect size was demonstrated for well-being, anxiety, and depression.

##### Virtual Delivery

There was a significant increase in well-being postintrevention (mean 50.50, SD 11.06) compared to preintervention (mean 39.50, SD 10.55; *t*_61_=7.18; Cohen *d_z_*=0.91, 95% CI 0.61-1.21; *P*<.001), emotional ties postintervention (mean 8.63, SD 1.99) compared to preintervention (mean 6.79, SD 2.54; *t*_61_=3.76; Cohen *d_z_*=0.48, 95% CI 0.21-0.74; *P*<.001), and social connectedness postintervention (mean 33.85, SD 11.33) compared to preintervention (mean 27.21, SD 11.69; *t*_61_=4.87; Cohen *d_z_*=0.62, 95% CI 0.34-0.89; *P*<.001). There was also a significant reduction in anxiety symptoms postintervention (mean 6.84, SD 5.18) compared to preintervention (mean 10.66, SD 5.17; *t*_61_=−5.37; Cohen *d_z_*=−0.68, 95% CI −0.95 to −0.40; *P*<.001) and depression symptoms postintervention (mean 7.40, SD 5.95) compared to preintervention (mean 11.94, SD 5.75; *t*_61_=−5.78; Cohen *d_z_=*−0.73*,* 95% CI −1.01 to −0.45; *P*<.001). A medium within-subjects effect was observed for pre-post change in emotional ties and social connectedness, and a large effect for change in well-being, depression, and anxiety symptoms.

### Objective 2: Comparing In-Person Versus Virtual Delivery on Within-Subject Change in Mental Ill Health Symptoms and Social Indices and Between-Group Differences

Descriptive statistics, including baseline between-group differences in delivery mode and means for study outcomes at T1 and T2, are presented in Multimedia Appendix 2. A significant baseline difference was observed for gender, with significantly fewer students identifying as women in the in-person delivery mode compared to the virtual delivery mode (*χ*^2^_1_=5.27, *P*=.02). No additional baseline differences in demographic characteristics or study outcomes were observed between delivery modes. Given the baseline difference in gender and the diagnostically heterogeneous sample, gender and mental illness were included as covariates in the 2×2 repeated-measures ANOVA. To maintain model parsimony, no additional demographic characteristics were included as covariates.

**Figure 1.**
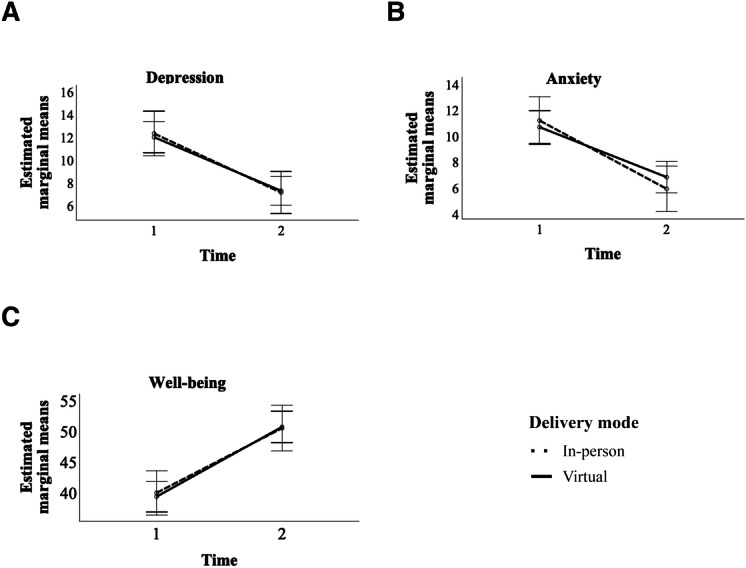
Change in mental ill health outcomes and well-being over time by delivery mode.

**Figure 2.**
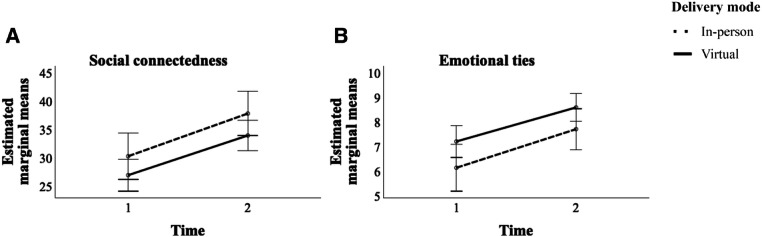
Change in social inclusion indices over time by delivery mode.

Results of the 2 (group: virtual, in-person delivery) × 2 (time: T1, T2) repeated-measures ANOVA, controlling for gender and mental illness, are presented in Table 3 and illustrated in Figure 1 for mental ill health symptoms and well-being, and Figure 2 for social inclusion indices. Error bars in Figures1  2 represent 95% CIs. There was a significant multivariate within-subjects effect of time. Univariate tests indicated significant reductions in mental ill health symptoms and improvements in social inclusion indices and well-being from T1 to T2, with a moderate effect for depression, well-being, social connectedness, and emotional ties, and a large effect for anxiety. There was no significant time × delivery mode, time × gender, or time × mental illness interaction effect. Multivariate between-subjects effects were significant for delivery mode. Univariate tests indicated that emotional ties significantly differed between delivery modes. Bonferroni-adjusted pairwise comparisons demonstrated an estimated marginal mean difference of −0.98 (SE 0.47), with lower emotional ties observed in the in-person delivery mode (mean 6.92, SE 0.39) compared to the virtual delivery mode (mean 7.90, SE 0.27). There were no significant multivariate between-subjects effects for gender or mental illness.

**Table 3.** Within- and between-subjects effects of the 2 × 2 repeated measures ANOVA (n=92).

Effect	Mean square	*F* test (*df*)	*ηp*²	*P* value
Within-subjects multivariate effects				
Time	—^a^	4.06 (5, 84)	0.20	.002
Time × delivery mode	—	0.72 (5, 84)	0.04	.60
Time × gender	—	0.87 (5, 84)	0.05	.50
Time × mental illness	—	1.39 (5, 84)	0.08	.24
Univariate effects of time^b^			
Depression	187.09	12.43 (1, 88)	0.12	<.001
Anxiety	232.39	17.03 (1, 88)	0.16	<.001
Well-being	841.95	13.48 (1, 88)	0.13	<.001
Social connectedness	584.77	11.57 (1, 88)	0.12	.001
Emotional ties	22.51	7.40 (1, 88)	0.08	.008
Between-subjects multivariate effects			
Delivery mode	—	2.49 (5, 84)	0.13	.04
Gender	—	0.42 (5, 84)	0.02	.84
Mental illness	—	2.16 (5, 84)	0.11	.07
Univariate effects of delivery mode^b^			
Depression	0.28	0.007	0.00	.93
Anxiety	1.47	0.04 (1, 88)	0.00	.84
Well-being	1.56	0.01 (1, 88)	0.00	.91
Social connectedness	492.30	2.74 (1, 88)	0.03	.10
Emotional ties	36.14	4.25 (1, 88)	0.05	.04

a Not applicable.

b Univariate effects are presented only for significant multivariate effects.

### Objective 3: The Unique Contribution of Mental Ill Health Symptoms and Social Inclusion Indices in Predicting Well-Being Change

Correlation coefficients among changes in the main study variables and demographic characteristics are presented in Table 4. Based on prior literature and evidence from bivariate change score correlations, gender, ethno-racial identity, and mental illness were included as covariates in the hierarchical regression analyses for virtual and in-person delivery [71-73].

**Table 4.** Pearson and Spearman bivariate correlation coefficients among change in mental ill health symptoms, social inclusion indices, well-being and demographic characteristics for in-person delivery sample 1 (n=87) and virtual delivery sample 2 (n=62).

		1	2	3	4	5	6	7	8	9
Gender										
	*r*	— ^a^^,b^	0.03	0.09	0.11	0.01	−0.06	0.07	0.23^c^	0.14
	*P* value	—	.77	.40	.34	.92	.60	.50	.03	.21
Mental illness										
	*r*	−0.21	—	−0.01	−0.17	−0.08	−0.02	−0.02	0.11	0.11
	*P* value	.11	—	.91	.13	.45	.86	.86	.33	.30
Age										
	*r*	−0.12	−0.05	—	0.12	0.10	0.04	0.11	−0.02	0.001
	*P* value	.34	.70	—	.30	.34	.72	.34	.89	.99
Ethno-racial identity										
	*r*	−0.16	0.38^d^	−0.13	—	−0.05	−0.05	0.11	−0.05	.17
	*P* value	.23	.003	.31	—	.68	.66	.33	.68	.12
Δ Anxiety										
	*r*	0.17	0.26^c^	0.03	0.30^c^	—	0.62^d^	−0.40^d^	−0.49^d^	−0.64^d^
	*P* value	.19	.04	.82	.02	—	<.001	<.001	<.001	<.001
Δ Depression										
	*r*	0.28^c^	0.06	0.11	0.18	0.75^d^	—	−0.39^d^	−0.54^d^	−0.68^d^
	*P* value	.03	.62	.38	.16	<.001	—	<.001	<.001	<.001
Δ Social^e^										
	*r*	−0.26	0.14	−0.24	−0.12	−0.54^d^	−0.68^d^	—	0.57^d^	0.51^d^
	*P* value	.04	.28	.06	.35	<.001	<.001	—	<.001	<.001
Δ Emotional ties										
	*r*	−0.19	−0.05	−0.10	0.04	−0.39^d^	−0.47^d^	0.35^d^	—	0.77^d^
	*P* value	.13	.72	.45	.76	.002	<.001	.006	—	<.001
Δ Well-being										
	*r*	−0.27	−0.16	−0.15	−0.09	−0.67^d^	−0.82^d^	0.69^d^	0.60^d^	—
	*P* value	.03	.22	.26	.48	<.001	<.001	<.001	<.001	—

a Correlation coefficients below the diagonal are for the virtual delivery sample, whereas correlation coefficients above the diagonal are for the in-person delivery sample.

b Not applicable.

c The correlation is significant at the .05 level (2-tailed).

d The correlation is significant at the .01 level (2-tailed).

e Social reflects social connectedness for virtual delivery and social relationship quality for in-person delivery.

#### In-Person Delivery

In the final model (Table 5), reductions in anxiety symptoms and depression symptoms pre-post program were associated with positive change in well-being, with a small effect size. Positive change in emotional ties was associated with positive change in well-being, with a medium effect size. Change in social relationship quality was not significantly associated with well-being change. Change in social inclusion indices contributed unique variance to predicting change in well-being above and beyond the contribution of mental ill health symptom change and demographic covariates. The final model predicted 72% of the variance in well-being change.

**Table 5.** Hierarchical linear regression predicting change in psychological well-being from demographic characteristics, mental ill health symptoms, and social inclusion indices for in-person delivery sample 1 (n=87) and virtual delivery sample 2 (n=62).

Variable	β	*R* ^2^_adj_	Δ*R*^2^	95% CI	*P* value
In-person delivery					
Step 1 (*F*_3,81_=1.66, *P*=.18)	—^a^	0.02	0.06	—	—
Gender	.15	—	—	−0.20 to 1.04	.18
Mental illness	.13	—	—	−0.26 to 1.02	.24
Ethno-racial identity	.15	—	—	−0.14 to 0.75	.18
Step 2 (*F*_5,79_=21.97, *P*<.001)	—	0.56	0.52	—	—
Gender	.11	—	—	−0.12 to 0.72	.16
Mental illness	.09	—	—	−0.18 to 0.70	.24
Ethno-racial identity	.10	—	—	−0.10 to 0.50	.19
Δ Anxiety	–.38	—	—	−0.58 to −0.20	<.001
Δ Depression	–.44	—	—	−0.62 to −0.25	<.001
Step 3 (*F*_7,77_=32.02, *P*<.001)	—	0.72	0.16	—	—
Gender	.02	—	—	−0.28 to 0.39	.75
Mental illness	.06	—	—	−0.17 to 0.52	.32
Ethno-racial identity	.11	—	—	−0.01 to 0.47	.06
Δ Anxiety	–.24	—	—	−0.41 to −0.09	.003
Δ Depression	–.26	—	—	−0.42 to −0.11	.002
Δ Social relationship quality	.02	—	—	−0.12 to 0.17	.75
Δ Emotional ties	.49	—	—	0.33 to 0.65	<.001
Virtual delivery					
Step 1 (*F*_3,58_=2.27, *P*=.08)	—	0.06	0.11	—	—
Gender	–.28	—	—	−2.48 to −0.15	.02
Mental illness	–.16	—	—	−0.86 to 0.21	.24
Ethno-racial identity	–.11	—	—	−0.79 to 0.33	.41
Step 2 (*F*_5,56_=25.82, *P*<.001)	—	0.67	0.59	—	—
Gender	.01	—	—	−0.69 to 0.79	.89
Mental illness	–.09	—	—	−0.50 to 0.16	.31
Ethno-racial identity	.11	—	—	−0.12 to 0.57	.20
Δ Anxiety	–.15	—	—	−0.12 to 0.57	.20
Δ Depression	–.73	—	—	−0.96 to −0.51	<.001
Step 3 (*F*_7,54_=27.74, *P*<.001)	—	0.75	0.08	—	—
Gender	.02	—	—	−0.55 to 0.73	.79
Mental illness	–.14	—	—	−0.57 to 0.03	.07
Ethno-racial identity	.07	—	—	−0.17 to 0.45	.36
Δ Anxiety	–.06	—	—	−0.26 to 0.15	.58
Δ Depression	–.49	—	—	−0.72 to −0.27	<.001
Δ Social connectedness	.27	—	—	0.08 to 0.45	.005
Δ Emotional ties	.25	—	—	0.10 to 0.40	.001

a Not applicable.

#### Virtual Delivery

In the final model (Table 5), depression symptom reduction was associated with positive change in well-being, with a medium effect size. Anxiety symptom reduction was not significantly associated with well-being change. Positive change in social connectedness and emotional ties was associated with positive change in well-being, with a small effect size. Positive change in social inclusion indices contributed to unique variance in well-being change above and beyond the contribution of mental ill health symptom change and demographic covariates. The final model predicted 75% of the variance in well-being change.

## Discussion

### Principal Findings

Using data from an ongoing and longstanding PA program tailored toward postsecondary student mental health (MoveU.HappyU), a comprehensive evaluation of the utility of in-person and virtual PA delivery for reducing mental ill health symptoms and promoting social inclusion was conducted. Findings from this study support the effectiveness of both in-person and virtual PA programming for reducing mental ill health symptoms (anxiety and depression) as well as promoting social inclusion indices (social connectedness, social relationship quality, and emotional ties) and well-being among postsecondary students. Reductions in mental ill health symptoms were associated with improved well-being, and improvements in social inclusion indices further contributed meaningfully to well-being change following program completion. The results provide support for the importance of further understanding intervention targets to address social inclusion indices alongside symptom reduction, indicating that PA programs can support both clinical and social dimensions of student mental health recovery.

Drawing on the broader mental health recovery literature [74,75], these findings support recovery-oriented approaches to mental health, which emphasize not only clinical recovery (ie, symptom reduction) but also social recovery (ie, rebuilding relationships, fostering belonging, and participating meaningfully in community life). Consistent with hypothesized objective 1 pre-post effects, in-person PA delivery was effective in improving social relationship quality and emotional ties with a medium effect, and in improving well-being and decreasing symptoms of anxiety and depression with a large effect. Similarly, virtual PA delivery led to improvements in emotional ties and social connectedness with a moderate effect, as well as improvements in well-being and reductions in anxiety and depression symptoms with a large effect. While PA interventions are often framed in clinical or behavioral health contexts, these results suggest that PA may also function as a social intervention by supporting students in rebuilding social networks and fostering a sense of social connectedness [25,76,77]. Notably, a substantial proportion of students were using primary forms of mental health treatment (eg, therapy and medication), suggesting that PA was often used alongside existing supports. This is consistent with models of care in which PA is positioned as a complementary component of mental health care and emphasizes that PA should be offered alongside primary treatments to support physical health, symptom management, and psychosocial functioning [24]. Future research is needed to better understand how PA can be integrated into postsecondary mental health care (eg, through social prescription and referral to community-based PA programs) to support mental health and social inclusion outcomes [24,78].

Aligning with the hypothesized results for objective 2*,* which examined whether intervention effects differed by delivery mode, there were no differences in pre-post change over time between virtual and in-person PA for reducing anxiety and depression symptoms and enhancing social connectedness, emotional ties, and well-being. These findings are consistent with prior evidence demonstrating beneficial effects of PA on mental ill health symptom reduction and well-being improvements with moderate-to-large effects [23,26,79]. These results further address the limited research examining social outcomes in PA-based mental health interventions [23,34,80], suggesting that improvements may also extend to social inclusion outcomes with moderate effects. These findings provide novel support for the mental health prevention value of virtual and in-person PA delivery, as limited research has examined whether mental health and social inclusion benefits differ based on delivery format [81]. However, given the quasi-experimental design of this study, further research is needed to replicate these findings using randomized controlled designs. Overall, future research is needed to better understand virtual delivery as an alternative to in-person PA delivery for students who may face barriers to in-person engagement due to geographic distance, personal preferences, scheduling conflicts, disability, or mental ill health–related challenges such as social anxiety [82-84].

In addition, the observed mental health and social inclusion benefits did differ based on mental illness diagnosis, despite a diagnostically heterogeneous sample, where a substantial proportion of students reported a past-year mental illness, supporting the transdiagnostic potential of PA programs (ie, defined as their capacity to address shared risk factors of mental illness across different mental illnesses and diagnostic criteria [85]). While evidence for the transdiagnostic potential of PA is promising, further research is needed to examine how program effects vary by diagnostic profiles and symptom severity within prevention-oriented and transdiagnostic approaches to better understand for whom PA interventions delivered virtually and in-person are most effective [85,86]. Practically, the transdiagnostic potential of PA may be particularly relevant in postsecondary contexts where students often present with a range of mental health concerns (diagnosed or undiagnosed) and where access to individualized, diagnosis-specific intervention is often limited [87,88].

Aligning with the hypothesized results for objective 3, reductions in mental ill health symptoms were associated with improved well-being, and changes in social inclusion indices contributed additional, meaningful variance in well-being change in both virtual and in-person PA delivery. In partial support of the hypotheses, positive change in emotional ties and social connectedness were associated with improvement in well-being in the virtual group, while only emotional ties, but not social relationship quality, were associated with well-being improvement in the in-person group. The inconsistent findings may reflect differences in the nature of the constructs [89,90]. Specifically, emotional ties and social connectedness reflect subjective experiences of closeness, belonging, and feeling emotionally supported, whereas social relationship quality represents a more general appraisal of satisfaction with one’s social relationships [90]. Researchers have suggested that feelings of closeness and connection may be more strongly associated with well-being than general assessments of one’s social life, relationships, and activities [89-91]. Further research is needed to explore how different dimensions of social inclusion uniquely contribute to well-being across PA delivery modes, with a practical implication for prioritizing PA program design elements (eg, peer PA mentors, group identity–building activities, and goal setting with social emphasis) that foster meaningful relationship building and social connectedness to support well-being [32,92,93].

In addition, reductions in depression symptoms were associated with improved well-being across in-person and virtual delivery. However, reductions in anxiety symptoms were only associated with improved well-being in the in-person group. The inconsistent results may reflect contextual factors related to global events during the virtual delivery period or inherent aspects of the virtual format. Specifically, virtual delivery occurred during the early stages of the COVID-19 pandemic, a time marked by heightened and persistent anxiety related to health concerns, uncertainty, and isolation [94,95]. These contextual stressors may have limited the extent to which reductions in anxiety translated into perceived improvements in well-being, even if symptoms decreased. From a theoretical perspective, virtual PA may be less effective in activating the mechanisms linked to well-being change and anxiety symptom reduction, such as real-world social interaction and opportunities to face anxiety-provoking situations in supportive environments, which may be more readily facilitated in face-to-face environments [96,97]. Future research should clarify whether virtual and in-person PA delivery activate different mechanistic pathways linking well-being change with symptom reduction and social inclusion improvements. In turn, future research focused on mechanisms of action could inform the optimization of virtual and in-person PA programs as a mental health prevention approach on postsecondary campuses.

### Limitations and Future Directions

Despite the strengths of this study, including a multiphase design and comparison of PA delivery modes, there are limitations that need to be acknowledged. Given the single-arm design, causal inferences are not possible, and it remains unknown whether the observed outcomes are directly attributable to the intervention or influenced by other factors such as the passage of time, external events, or participant heterogeneity across intervention phases. For example, individual variability in workload fluctuations, mental ill health symptom severity, and concurrent treatment were not accounted for, and future research using person-centered data analysis approaches is needed to account for the effects of within-person variability [98]. Moreover, while program outcomes over time did not differ based on gender, the samples across the intervention phases predominantly identified as women, which is a limitation consistent with the broader literature [23]. Further research should explore strategies to engage men and assess whether engagement and intervention outcomes differ among underserved student groups (eg, racialized, 2SLGBTQ+, and students living with a disability) to ensure PA programs are effective, inclusive, and accessible in their reach and impact [99,100]. Furthermore, data collection during virtual delivery occurred in the early stages of the COVID-19 pandemic, which may limit the generalizability of the findings due to contextual stressors such as heightened health-related anxiety, uncertainty, and social isolation [94,95]. Finally, this study only examined social inclusion indices related to social relationships and social connectedness. Further research is needed to understand the role of systemic social determinants of health in lifestyle intervention research, including safe and stable housing, access to services, and food and financial security [101].

### Conclusions

Mental health recovery and treatment are often conceptualized and measured primarily through symptom reduction, with insufficient consideration of social recovery, a limitation that is similarly reflected in the PA and mental health literature [23,74]. This study contributes novel insights into the effectiveness of a 6-week one-on-one supervised in-person and virtual PA program for reducing symptoms of mental ill health and supporting social inclusion indices among postsecondary students. In postsecondary contexts where loneliness and social disconnection are increasingly prevalent, PA interventions that promote social inclusion and reduce symptoms of mental ill health may play a critical role in supporting student well-being and reducing vulnerability to a range of mental ill health concerns [102]. In addition, with the rise in distance-based learning on postsecondary campuses, these findings provide practical implications for further understanding the effectiveness of virtually delivered PA as an alternative delivery format to in-person PA, particularly among students who face barriers to in-person engagement. Overall, the findings provide support for the effectiveness of virtual and in-person PA delivery for targeting shared risk factors (eg, low social connectedness and poor psychological well-being) that contribute to mental ill health across clinical and nonclinical populations.

## Supplementary material

10.2196/92076 Multimedia Appendix 1 Participant flow across the intervention phases.

10.2196/92076 Multimedia Appendix 2 Demographic and baseline outcome differences between in-person and virtual delivery.

## Abbreviations

GAD-7: Generalized Anxiety Disorder-7
MHI-38: Mental Health Index-38
MPARC: Mental Health and Physical Activity Research Centre
MVPA: moderate-to-vigorous physical activity
PA: physical activity
PHQ-9: Patient Health Questionnaire-9
RCT: randomized controlled trial
REDCap: Research Electronic Data Capture

## References

[1] Arnett JJ, Murray JL, Arnett JJ (2019). Emerging Adulthood and Higher Education.

[2] Patel V, Saxena S, Lund C (2018). The Lancet Commission on global mental health and sustainable development. Lancet.

[3] Curtis A, Bearden A, Turner JP (2023). Post‐secondary student transitions and mental health: literature review and synthesis. New Dir High Educ.

[4] Murray JL, Murray JL, Arnett JJ (2018). Emerging Adulthood and Higher Education: A New Student Development Paradigm.

[5] Kwan MY, Cairney J, Faulkner GE, Pullenayegum EE (2012). Physical activity and other health-risk behaviors during the transition into early adulthood: a longitudinal cohort study. Am J Prev Med.

[6] Demenech LM, Duffy A (2026). Student mental health is in crisis — here’s how to help. Nature New Biol.

[7] Fruehwirth JC, Mazzolenis ME, Pepper MA, Perreira KM (2023). Perceived stress, mental health symptoms, and deleterious behaviors during the transition to college. PLoS One.

[8] Duffy A, Keown-Stoneman C, Goodday S (2020). Predictors of mental health and academic outcomes in first-year university students: identifying prevention and early-intervention targets. BJPsych Open.

[9] Ogrodniczuk JS, Kealy D, Laverdière O (2021). Who is coming through the door? A national survey of self‐reported problems among post‐secondary school students who have attended campus mental health services in Canada. Couns Psychother Res.

[10] Long LD (2025). Healthy habits, healthy minds: an exploration of lifestyle behaviors and mental health among college students. J Am Coll Health.

[11] (2015). The Okanagan Charter. The University of British Columbia.

[12] National standard for mental health and well-being for post-secondary students. Mental Health Commission of Canada.

[13] Garrod N, Macfarlane B (2007). Scoping the duals: structural challenges of combining further and higher education in post‐secondary institutions. High Educ Q.

[14] Dooris M, Powell S, Farrier A (2020). Conceptualizing the “whole university” approach: an international qualitative study. Health Promot Int.

[15] McGorry PD, Mei C, Dalal N (2024). The Lancet Psychiatry Commission on youth mental health. Lancet Psychiatry.

[16] (2022). World mental health report: transforming mental health for all. World Health Organization.

[17] Fusar-Poli P, Correll CU, Arango C, Berk M, Patel V, Ioannidis JPA (2021). Preventive psychiatry: a blueprint for improving the mental health of young people. World Psychiatry.

[18] Barnett P, Arundell LL, Saunders R, Matthews H, Pilling S (2021). The efficacy of psychological interventions for the prevention and treatment of mental health disorders in university students: a systematic review and meta-analysis. J Affect Disord.

[19] deJonge ML, Ashdown-Franks G, Sabiston CM, Zangeneh M, Nouroozifar N, Chou P (2022). Post-Secondary Education Mental Health: A Global Perspective.

[20] Jeftic I, Furzer BJ, Dimmock JA (2023). Structured exercise programs for higher education students experiencing mental health challenges: background, significance, and implementation. Front Public Health.

[21] Cunningham CE, Zipursky RB, Christensen BK (2017). Modeling the mental health service utilization decisions of university undergraduates: a discrete choice conjoint experiment. J Am Coll Health.

[22] Litwiller F, White C, Hamilton-Hinch B, Gilbert R (2022). The impacts of recreation programs on the mental health of postsecondary students in North America: an integrative review. Leis Sci.

[23] Donnelly S, Penny K, Kynn M (2024). The effectiveness of physical activity interventions in improving higher education students’ mental health: a systematic review. Health Promot Int.

[24] Deenik J, Vermeulen JM, Teasdale SB (2025). Lifestyle psychiatry: a conceptual framework for application in mental healthcare and support. BMJ Ment Health.

[25] Teasdale SB, Machaczek KK, Marx W (2025). Implementing lifestyle interventions in mental health care: third report of the Lancet Psychiatry Physical Health Commission. Lancet Psychiatry.

[26] Huang K, Beckman EM, Ng N (2024). Effectiveness of physical activity interventions on undergraduate students’ mental health: systematic review and meta-analysis. Health Promot Int.

[27] Chatoor K, Pilla N, Balata L, Shah H, Kaufman A Supporting student mental health in ontario: exploring best practices and identifying gaps. Higher Education Quality Council of Ontario.

[28] Sampson K, Priestley M, Dodd AL (2022). Key questions: research priorities for student mental health. BJPsych Open.

[29] Filia K, Jackson H, Cotton S, Killackey E (2019). Understanding what it means to be socially included for people with a lived experience of mental illness. Int J Soc Psychiatry.

[30] deJonge ML, Sabiston CM, Hamza CA, Darnell SC (2025). Modeling associations between physical recreation engagement and correlates of post-secondary student psychosocial well-being: exploring differences among students living with and without a mental health condition. Psychol Sport Exerc.

[31] Litwiller F, White C, Gallant KA (2017). The benefits of recreation for the recovery and social inclusion of individuals with mental illness: an integrative review. Leis Sci.

[32] Healy LC, Benkwitz A, McVinnie Z (2023). Embedding physical activity into community-based peer support groups for those severely affected by mental illness. Int J Environ Res Public Health.

[33] Priestley M, Broglia E, Hughes G, Spanner L (2022). Student perspectives on improving mental health support services at university. Couns Psychother Res.

[34] Kemel PN, Porter JE, Coombs N (2022). Improving youth physical, mental and social health through physical activity: a Systematic literature review. Health Promot J Austr.

[35] Smith N, Graham JM, Waddell-Henowitch C, De Moissac D, Lam M (2023). Post-secondary student belonging in a virtual learning environment during COVID-19. Can J High Educ.

[36] Palvia S, Aeron P, Gupta P (2018). Online education: worldwide status, challenges, trends, and implications. J Glob Inf Technol Manag.

[37] Johnson N, Seaman J (2021). The growth of online learning and digital learning resources in Canadian post-secondary education. https://www.cdlra-acrfl.ca/wp-content/uploads/2022/04/2021_special-topics_en.pdf.

[38] Kimhy D, Ospina LH, Wall M (2025). Telehealth-based vs in-person aerobic exercise in individuals with schizophrenia: comparative analysis of feasibility, safety, and efficacy. JMIR Ment Health.

[39] Browne J, Naslund JA, Salwen-Deremer JK, Sarcione C, Cabassa LJ, Aschbrenner KA (2024). Factors influencing engagement in in‐person and remotely delivered lifestyle interventions for young adults with serious mental illness: a qualitative study. Early Interv Psychiatry.

[40] Rosenbaum S, Newby JM, Steel Z, Andrews G, Ward PB (2015). Online physical activity interventions for mental disorders: a systematic review. Internet Interv.

[41] Bhundoo AK, Pillay JD, Wilke J (2025). The effectiveness of online exercise on physical activity, motor function, and mental health: systematic review and meta-analysis. J Med Internet Res.

[42] LaMonica HM, Hickie IB, Capon W (2025). Digital tools to support post-secondary student mental health and wellbeing. Early Interv Psychiatry.

[43] Powers SL, Wilson OWA, Bopp M (2022). Challenges faced and solutions implemented in response to the COVID-19 pandemic among North American college campus recreation staff. Recreat Sports J.

[44] Keyes CLM (1998). Social well-being. Soc Psychol Q.

[45] deJonge ML, Omran J, Faulkner GE, Sabiston CM (2020). University students’ and clinicians’ beliefs and attitudes towards physical activity for mental health. Ment Health Phys Act.

[46] deJonge ML, Jain S, Faulkner GE, Sabiston CM (2021). On campus physical activity programming for post-secondary student mental health: examining effectiveness and acceptability. Ment Health Phys Act.

[47] Czajkowski SM, Powell LH, Adler N (2015). From ideas to efficacy: the ORBIT model for developing behavioral treatments for chronic diseases. Health Psychol.

[48] deJonge ML, Yuen S, Simard L, Sabiston CM (2025). One-on-one and group-based physical activity intervention compared to a waitlist control for post-secondary student mental health and social well-being: a 3-arm parallel randomized controlled trial protocol. PLoS One.

[49] Veit CT, Ware JE (1983). The structure of psychological distress and well-being in general populations. J Consult Clin Psychol.

[50] Rush AJ, South CC, Jha MK, Grannemann BD, Trivedi MH (2019). Toward a very brief quality of life enjoyment and Satisfaction Questionnaire. J Affect Disord.

[51] Kroenke K, Spitzer RL, Williams JBW (2001). The PHQ-9: validity of a brief depression severity measure. J Gen Intern Med.

[52] Spitzer RL, Kroenke K, Williams JBW, Löwe B (2006). A brief measure for assessing generalized anxiety disorder: the GAD-7. Arch Intern Med.

[53] Lee RM, Robbins SB (1995). Measuring belongingness: the social connectedness and the social assurance scales. J Couns Psychol.

[54] Lederman O, Suetani S, Stanton R (2017). Embedding exercise interventions as routine mental health care: implementation strategies in residential, inpatient and community settings. Australas Psychiatry.

[55] Michie S, Richardson M, Johnston M (2013). The behavior change technique taxonomy (v1) of 93 hierarchically clustered techniques: building an international consensus for the reporting of behavior change interventions. Ann Behav Med.

[56] Vella SA, Aidman E, Teychenne M (2023). Optimising the effects of physical activity on mental health and wellbeing: a joint consensus statement from Sports Medicine Australia and the Australian Psychological Society. J Sci Med Sport.

[57] Orfaly RA, Frances JC, Campbell P, Whittemore B, Joly B, Koh H (2005). Train-the-trainer as an educational model in public health preparedness. J Public Health Manag Pract.

[58] Harris PA, Taylor R, Minor BL (2019). The REDCap consortium: building an international community of software platform partners. J Biomed Inform.

[59] (2017). Global action plan on physical activity 2018-2030: more actions people for a healthier world. World Health Organization.

[60] Godin G (2011). The Godin-Shephard Leisure-Time Physical Activity Questionnaire. Health Fit J Canada.

[61] Amireault S, Godin G (2015). The Godin-Shephard Leisure-Time Physical Activity Questionnaire: validity evidence supporting its use for classifying healthy adults into active and insufficiently active categories. Percept Mot Skills.

[62] Morgan JF, Reid F, Lacey JH (1999). The SCOFF questionnaire: assessment of a new screening tool for eating disorders. BMJ.

[63] Warburton DER, Jamnik VK, Bredin SSD, Gledhill N (2011). The Physical Activity Readiness Questionnaire for Everyone (PAR-Q+) and electronic Physical Activity Readiness Medical Examination (ePARmed-X+). Health Fit J Canada.

[64] McNeish D (2018). Thanks coefficient alpha, we’ll take it from here. Psychol Methods.

[65] Craig CL, Marshall AL, Sjöström M (2003). International Physical Activity Questionnaire: 12-country reliability and validity. Med Sci Sports Exerc.

[66] Best H, Wolf C (2014). The SAGE Handbook of Regression Analysis and Causal Inference.

[67] Sawyer SF (2009). Analysis of variance: the fundamental concepts. J Man Manip Ther.

[68] Lovakov A, Agadullina ER (2021). Empirically derived guidelines for effect size interpretation in social psychology. Euro J Social Psych.

[69] Nieminen P (2022). Application of standardized regression coefficient in meta-analysis. BioMedInformatics.

[70] Richardson JTE (2011). Eta squared and partial eta squared as measures of effect size in educational research. Educ Res Rev.

[71] Hale GE, Colquhoun L, Lancastle D, Lewis N, Tyson PJ (2021). Review: physical activity interventions for the mental health and well-being of adolescents - a systematic review. Child Adolesc Ment Health.

[72] Huang CY, Zane N (2016). Cultural influences in mental health treatment. Curr Opin Psychol.

[73] Przybylko G, Morton D, Morton J, Renfrew M (2021). The influence of gender and age on the outcomes of and adherence to a digital interdisciplinary mental health promotion intervention in an Australasian nonclinical setting: cohort study. JMIR Ment Health.

[74] Barbic SP, Ow N, Kidd SA (2025). Optimizing measurement potential in mental health clinical practice: The Canadian Personal Recovery Outcome Measure (C-PROM) study. J Psychosoc Rehabil Ment Health.

[75] Dell NA, Long C, Mancini MA (2021). Models of mental health recovery: an overview of systematic reviews and qualitative meta-syntheses. Psychiatr Rehabil J.

[76] Stubbs B, Ma R, Schuch F (2024). Physical activity and mental health: a little less conversation, a lot more action. J Phys Act Health.

[77] Herbert C (2022). Enhancing mental health, well-being and active lifestyles of university students by means of physical activity and exercise research programs. Front Public Health.

[78] Chatterjee HJ, Camic PM, Lockyer B, Thomson LJM (2018). Non-clinical community interventions: a systematised review of social prescribing schemes. Arts Health.

[79] Chen Z, Huang H, Liu R, Tang Z (2024). Effects of internet-based exercise intervention on depression and anxiety: a systematic review and meta-analysis. Medicine (Baltimore).

[80] Pascoe M, Bailey AP, Craike M (2020). Physical activity and exercise in youth mental health promotion: a scoping review. BMJ Open Sport Exerc Med.

[81] Vella SA, Sutcliffe JT, Fernandez D (2023). Context matters: a review of reviews examining the effects of contextual factors in physical activity interventions on mental health and wellbeing. Ment Health Phys Act.

[82] Goldstein SP, Forman EM, Butryn ML, Herbert JD (2018). Differential programming needs of college students preferring web-based versus in-person physical activity programs. Health Commun.

[83] Matthews N, Seaman R, Bremer E (2023). Program evaluation of a virtual physical activity program for individuals with disabilities. Front Sports Act Living.

[84] Tovin MM, Núñez-Gaunaurd A (2024). Implementation of peer-assisted physical activity via telehealth for adults on the autism spectrum: a mixed methods feasibility study. Phys Ther.

[85] Solmi M, Basadonne I, Bodini L (2025). Exercise as a transdiagnostic intervention for improving mental health: an umbrella review. J Psychiatr Res.

[86] White RL, Vella S, Biddle S (2024). Physical activity and mental health: a systematic review and best-evidence synthesis of mediation and moderation studies. Int J Behav Nutr Phys Act.

[87] Burke AS, Shapero BG, Pelletier-Baldelli A (2019). Rationale, methods, feasibility, and preliminary outcomes of a transdiagnostic prevention program for at-risk college students. Front Psychiatry.

[88] Moghimi E, Stephenson C, Gutierrez G (2023). Mental health challenges, treatment experiences, and care needs of post-secondary students: a cross-sectional mixed-methods study. BMC Public Health.

[89] Reis HT, Ryff CD, Singer B (2001). Emotion, Social Relationships and Health.

[90] Ruppel EH, Child S, Fischer CS, Botchway M (2022). Distinct aspects of human connection associated with subjective well-being. SSM Ment Health.

[91] Allen KA, Kern ML, Rozek CS, McInereney D, Slavich GM (2021). Belonging: a review of conceptual issues, an integrative framework, and directions for future research. Aust J Psychol.

[92] Glazzard J, Rose A, Ogilvie P (2021). The impact of peer mentoring on students’ physical activity and mental health. J Public Ment Health.

[93] Li Z, Li J, Kong J, Li Z, Wang R, Jiang F (2024). Adolescent mental health interventions: a narrative review of the positive effects of physical activity and implementation strategies. Front Psychol.

[94] Dey P, De Souza LR (2025). Public health challenges for post-secondary students during COVID-19: a scoping review. Community Health Equity Res Pol.

[95] Ewing L, Hamza CA, Walsh K, Goldstein AL, Heath NL (2022). A qualitative investigation of the positive and negative impacts of the COVID-19 pandemic on post-secondary students’ mental health and well-being. Emerg Adulthood.

[96] Kroencke L, Harari GM, Back MD, Wagner J (2023). Well-being in social interactions: examining personality-situation dynamics in face-to-face and computer-mediated communication. J Pers Soc Psychol.

[97] Liang N, Grayson SJ, Kussman MA, Mildner JN, Tamir DI (2024). In-person and virtual social interactions improve well-being during the COVID-19 pandemic. Comput Human Behav Rep.

[98] Compton MT, Shim RS (2020). Mental illness prevention and mental health promotion: when, who, and how. Psychiatr Serv.

[99] Cleverley K, Salman S, Davies J (2025). Frameworks used to engage postsecondary students in campus mental health research: a scoping review. Health Expect.

[100] Taylor ME, Liu M, Abelson S, Eisenberg D, Lipson SK, Schueller SM (2024). The reach, effectiveness, adoption, implementation, and maintenance of digital mental health interventions for college students: a systematic review. Curr Psychiatry Rep.

[101] Gardner A, Filia K, Killackey E, Cotton S (2019). The social inclusion of young people with serious mental illness: a narrative review of the literature and suggested future directions. Aust N Z J Psychiatry.

[102] Priestley M, Hall A, Wilbraham SJ, Mistry V, Hughes G, Spanner L (2022). Student perceptions and proposals for promoting wellbeing through social relationships at university. J Furth High Educ.

