# Supplementary material for: Examining the Social and Mental Health Benefits of Virtual and In-Person Physical Activity Intervention Among Postsecondary Students: Quasi-Experimental Study
Source: JMIR Ment Health. 2026 Jun 11;13:e92076. doi: 10.2196/92076 (PMC13257782; doi:10.2196/92076)
Supplement: Multimedia Appendix 1 [file mental-v13-e92076-s001.docx]

**Multimedia Appendix 1**

**Participant Flow Across the Intervention Phases**

Participant flow charts for Phase 1, Phase 2, and Phase 3 data collection cycles are presented in Figures S1-S3, respectively.


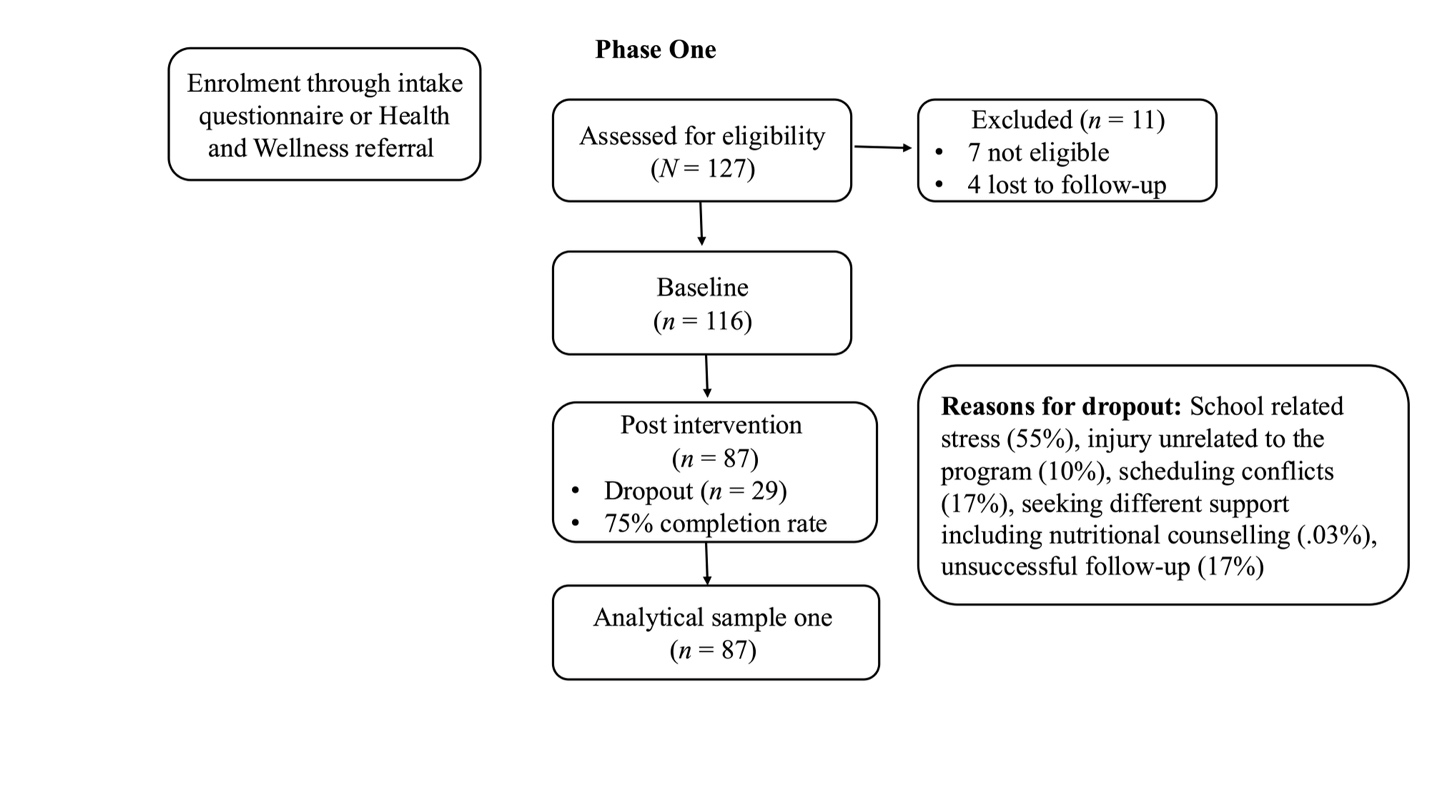


**Figure S1.** Phase 1 in-person delivery participant flow. ​


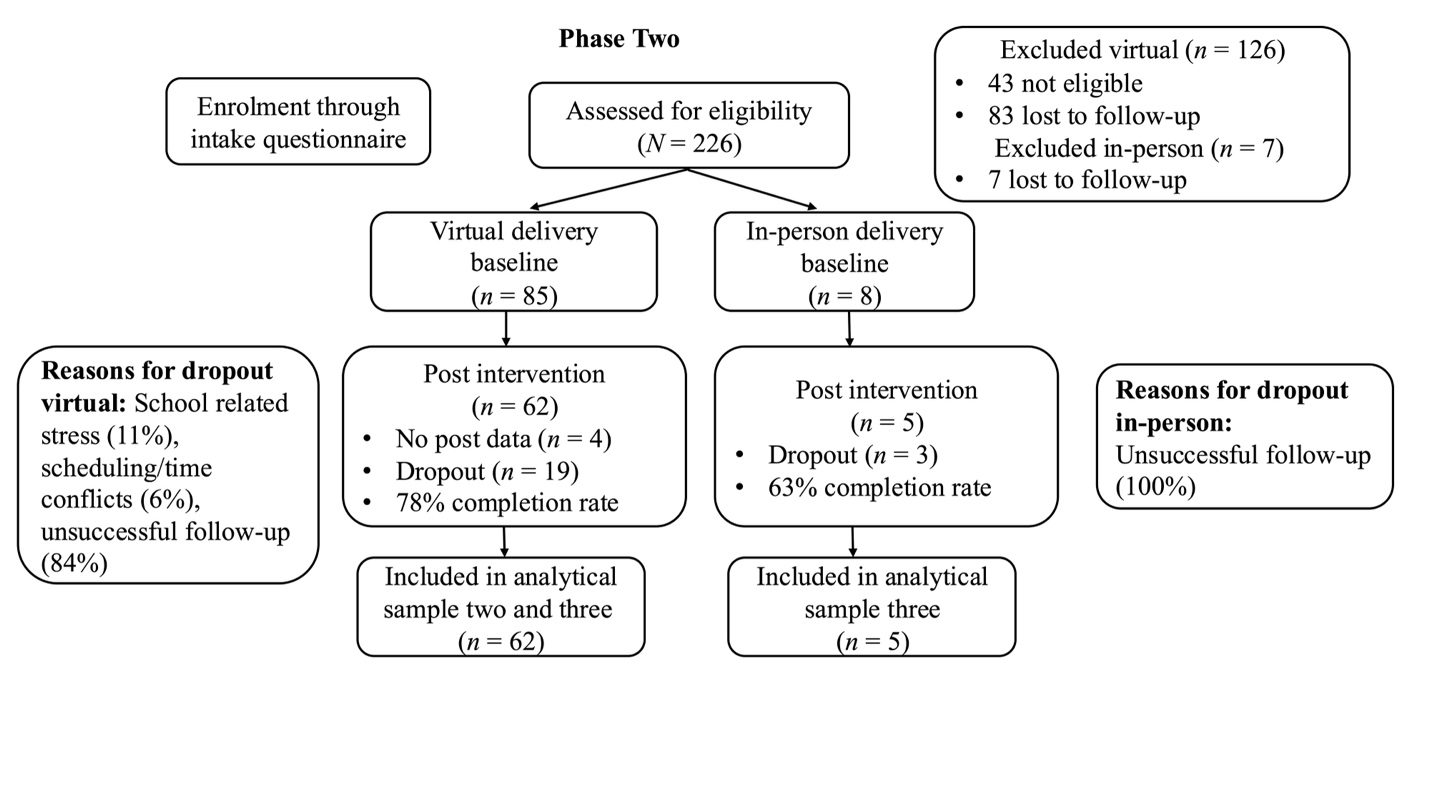
**Figure S2.** Phase 2 in-person and virtual delivery participant flow. ​

**
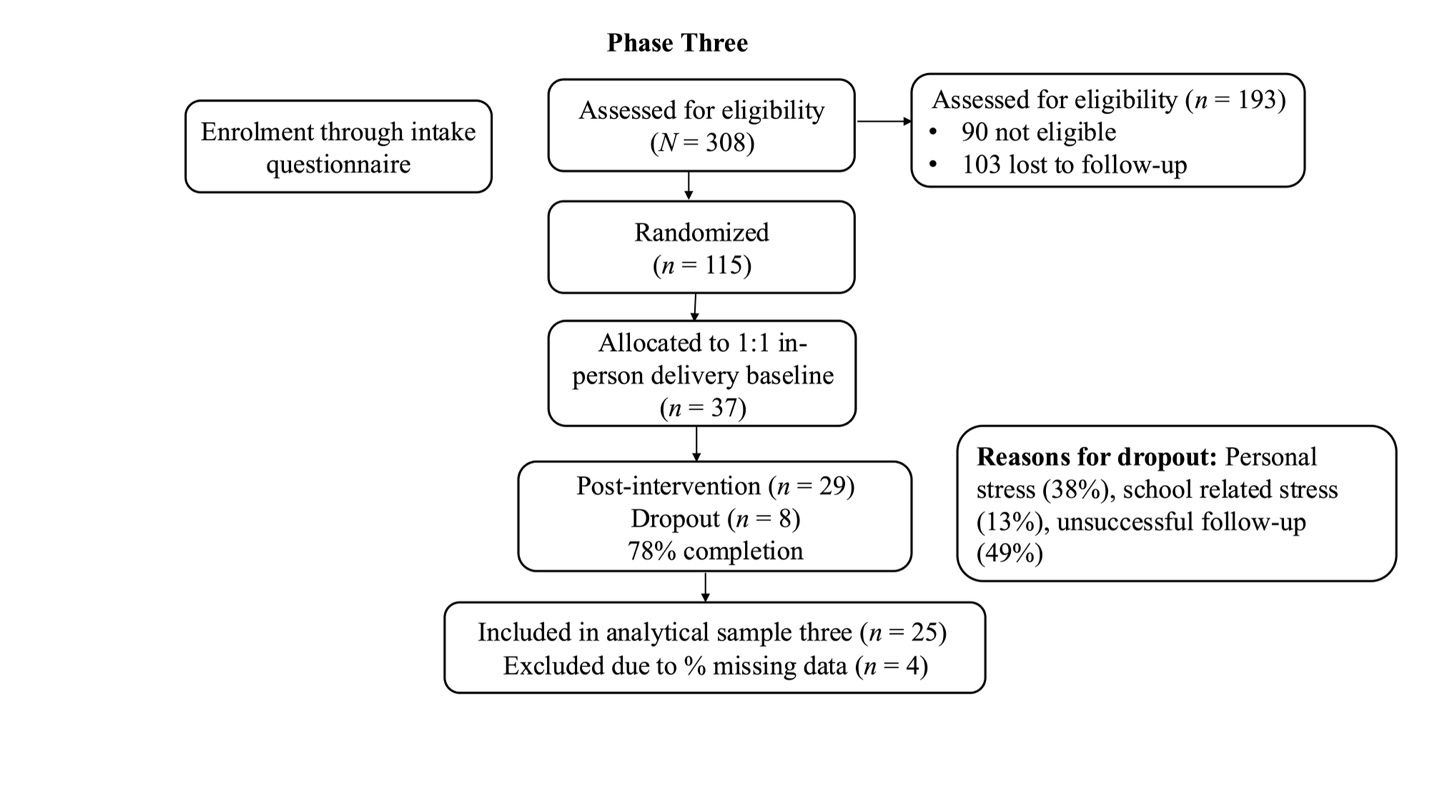
**

**Figure S3.** *Phase Three* in-person delivery participant flow. Participants were excluded with % missing data > 15 %.
