# Supplementary material for: Examining the Social and Mental Health Benefits of Virtual and In-Person Physical Activity Intervention Among Postsecondary Students: Quasi-Experimental Study
Source: JMIR Ment Health. 2026 Jun 11;13:e92076. doi: 10.2196/92076 (PMC13257782; doi:10.2196/92076)
Supplement: Multimedia Appendix 2 [file mental-v13-e92076-s002.docx]

**Multimedia Appendix 2**

**Demographic and Baseline Outcome Differences Between In-Person and Virtual Delivery**

Descriptive statistics for demographic characteristics and main variables at T2 and T2 stratified by delivery mode are presented in Table S1. There was a significant between group difference for gender [*χ2* (1) = 5.27, *P* = .02], with in-person delivery containing significantly less women compared to virtual delivery. There were no significant between group differences at baseline for demographic characteristics including age [*χ2* (1) = 0.28, *P* = .60], ethno-racial identity [*χ2* (1) = 0.49, *P* = .49], and mental illness diagnosis [*χ2* (1) = 1.17, *P* = .28].

There were no between group differences in baseline well-being for in-person delivery [*M (SD)* = 39.77 (9.04)] compared to virtual delivery [*M (SD)* = 39.35 (10.16)], *t* (90) = -0.19, *P* = .86, *d* = -0.04 [-0.48, 0.39]; depression for in-person delivery [*M (SD)* = 12.43 (4.44)] compared to virtual delivery [*M (SD)* = 11.94 (5.75)], *t* (90) = -0.42, *P* = .68, *d* = -0.09 [-0.53, 0.34]; anxiety for in-person delivery [*M (SD)* = 11.23 (4.38)] compared to virtual delivery [*M (SD)* = 10.66 (5.17)], *t* (90) = -0.52, *P* = .60, *d* = -0.12 [-0.55, 0.32]; social connectedness for in-person delivery [*M (SD)* = 29.77 (9.32)] compared to virtual delivery [*M (SD)* = 27.21 (11.69)], *t* (90) = -1.05, *P* = .30, *d = -*0*.23* [0.67, 0.21]; and emotional ties for in-person delivery [*M (SD)* = 6.13 (2.51)] compared to virtual delivery [*M (SD)* = 7.21 (2.54)], *t* (90) = 1.91, *P* = .06, *d* = 0.43 [-0.02, 0.87].

| **Table S1.** Descriptive statistics for baseline demographics and study outcomes at T2 and T2 by virtual (*n* = 62) and in-person delivery (*n* = 30) for analytical sample three (*N* = 92) | | |
| --- | --- | --- |
| **Variables** | **In-Person Delivery** | **Virtual Delivery** |
| **Demographic characteristics** |  |  |
| **Gender, n (%)** |  |  |
| Women | 24 (80%) | 59 (95%) |
| Non-women^a^ | 6 (20%) | 3 (5%) |
| **Mental illness, n (%)** |  |  |
| Diagnosis | 20 (67%) | 34 (55%) |
| No diagnosis | 10 (33%) | 28 (45%) |
| **Age, n (%)** |  |  |
| Young adult | 19 (63%) | 42 (69%) |
| Adult | 11 (37%) | 19 (31%) |
| **Ethno-racial identity, n (%)** |  |  |
| White | 8 (27%) | 21 (34%) |
| Less represented groups^b^ | 22 (73%) | 41 (66%) |
| **Study Outcomes, mean (SD)** |  |  |
| Anxiety pre | 11.23 (4.38) | 10.66 (5.17) |
| Anxiety post | 5.87 (4.18) | 6.84 (5.18) |
| Depression pre | 12.43 (4.44) | 11.94 (5.74) |
| Depression post | 7.03 (3.58) | 7.31 (5.69) |
| Social connectedness pre | 29.77 (9.32) | 27.21 (11.69) |
| Social connectedness post | 38.03 (8.24) | 33.85 (11.33) |
| Emotional ties pre | 6.13 (2.52) | 7.21 (2.54) |
| Emotional ties post | 7.70 (2.52) | 8.60 (2.08) |
| Well-being pre | 39.77 (9.04) | 39.35 (10.16) |
| Well-being post | 50.43 (9.67) | 50.63 (10.72) |
| ^a^ For in-person delivery non-women included non-binary 2 (7%) and Men 4 (13%); For virtual delivery non-women included non-binary 3 (5%)  ^b^ For in-person delivery less-represented groups included: Black 2 (7%); Chinese 7 (23%); Japanese 1 (3%); Korean 1 (3%); South Asian 5 (17%); West Asian 1 (3%); Latin American 1 (3%); Population group not listed 1 (3%); Multiple groups selected 3 (10%); For virtual delivery less-represented groups included: Black 2 (3%); Chinese 10 (16%); Korean 3 (5%); South Asian 13 (21%); Southeast Asian 2 (3%); West Asian 3 (5%); Latin American 1 (2%); Population group not listed 1 (2%); Multiple groups selected 6 (10%) | | |
